# More concerns on triphala mouthwash

**DOI:** 10.4103/0974-7788.72498

**Published:** 2010

**Authors:** Viroj Wiwanitkit

**Affiliations:** *Wiwanitkit House, Bangkhae, Bangkok, Thailand 10160, E-mail: wviroj@yahoo.com*

Sir,

I read with great interest a recent published work on triphala mouthwash by Tandon *et al*[1] They concluded that there was no significant difference between *triphala* and chlorhexidine mouthwashes.[1] I agree that this work confirms the dental usefulness of triphala. The previous report also mentioned the counteraction on biofilm formed on tooth substrate.[2] However, there are some facts on the triphala to be known. Triphala has been recently reported to have an *in vitro* effect on drug modulating enzymes.[3] Disturbance on cytochrome P450 is mentioned, thereby the likelihood of herb–drug interactions, if these are administered concomitantly, can be expected.[3]
